# Addressing challenges for clinical research responses to emerging epidemics and pandemics: a scoping review

**DOI:** 10.1186/s12916-020-01624-8

**Published:** 2020-06-25

**Authors:** Louise Sigfrid, Katherine Maskell, Peter G. Bannister, Sharif A. Ismail, Shelui Collinson, Sadie Regmi, Claire Blackmore, Eli Harriss, Kajsa-Stina Longuere, Nina Gobat, Peter Horby, Mike Clarke, Gail Carson

**Affiliations:** 1 Centre for Tropical Medicine and Global Health, Nuffield Department of Medicine, University of Oxford, New Richards Building, Old Road Campus, Oxford, OX3 7LG UK; 2 Deparment for Primary Care and Public Health, Brighton and Sussex Medical School, Brighton, UK; 3 Department of Global Health and Development, London School of Hygiene and Tropical Medicine, London, UK; 4 School of Population Health and Environmental Sciences, King’s College London, London, UK; 5 Department of Primary Care and Public Health, Imperial College London, London, UK; 6 University Hospitals of Derby and Burton NHS Foundation Trust, Derby, UK; 7 Bodleian Health Care Libraries, University of Oxford, Oxford, UK; 8 Nuffield Dep of Primary Care Health Sciences, University of Oxford, Oxford, UK; 9 Evidence Aid, Centre for Public Health, Queen’s University Belfast, Belfast, UK

**Keywords:** Challenges, Barriers, Solutions, Facilitators, Clinical research, Emerging infectious diseases, Epidemic, Pandemic, Preparedness

## Abstract

**Background:**

Major infectious disease outbreaks are a constant threat to human health. Clinical research responses to outbreaks generate evidence to improve outcomes and outbreak control. Experiences from previous epidemics have identified multiple challenges to undertaking timely clinical research responses. This scoping review is a systematic appraisal of political, economic, administrative, regulatory, logistical, ethical and social (PEARLES) challenges to clinical research responses to emergency epidemics and solutions identified to address these.

**Methods:**

A scoping review. We searched six databases (MEDLINE, Embase, Global Health, PsycINFO, Scopus and Epistemonikos) for articles published from 2008 to July 2018. We included publications reporting PEARLES challenges to clinical research responses to emerging epidemics and pandemics and solutions identified to address these. Two reviewers screened articles for inclusion, extracted and analysed the data.

**Results:**

Of 2678 articles screened, 76 were included. Most presented data relating to the 2014–2016 Ebola virus outbreak or the H1N1 outbreak in 2009. The articles related to clinical research responses in Africa (*n* = 37), Europe (*n* = 8), North America (*n* = 5), Latin America and the Caribbean (*n* = 3) and Asia (*n* = 1) and/or globally (*n* = 22). A wide range of solutions to PEARLES challenges was presented, including a need to strengthen global collaborations and coordination at all levels and develop pre-approved protocols and equitable frameworks, protocols and standards for emergencies. Clinical trial networks and expedited funding and approvals were some solutions implemented. National ownership and community engagement from the outset were a key enabler for delivery. Despite the wide range of recommended solutions, none had been formally evaluated.

**Conclusions:**

To strengthen global preparedness and response to the COVID-19 pandemic and future epidemics, identified solutions for rapid clinical research deployment, delivery, and dissemination must be implemented. Improvements are urgently needed to strengthen collaborations, funding mechanisms, global and national research capacity and capability, targeting regions vulnerable to epidemics and pandemics. Solutions need to be flexible to allow timely adaptations to context, and research led by governments of affected regions. Research communities globally need to evaluate their activities and incorporate lessons learnt to refine and rehearse collaborative outbreak response plans in between epidemics.

## Background

Clinical research forms the basis for evidence-based clinical management of patients and can contribute to effective outbreak control. Although activities have improved collective preparedness to respond to public health emergencies [1, 2], experiences from previous outbreaks have highlighted many ongoing challenges for clinical research responses to epidemics [3, 4]. Some of these stem from the inherently unpredictable nature of emerging infections. Epidemics occur intermittently across geopolitical and cultural boundaries. Some can be forecast, and others emerge unexpectedly and disproportionally affect resource-poor settings with fragile healthcare systems and infrastructure, adding additional challenges to responses [5]. Previous epidemics have generated important information that has helped inform preparedness and response; however, it has also highlighted systemic challenges to our global capability to address important clinical research questions in these environments [1]. Clinical research takes time to plan, conduct and disseminate, a luxury that is rarely available during an outbreak. Ethical and regulatory frameworks designed for non-acute epidemics are not necessarily fit for the purpose of acute epidemic research [6, 7]. Conducting research under emergency conditions requires agility, intense activity, flexibility and adaptability to context [3, 4].

The aim of this scoping review is to identify how challenges to delivering essential clinical research during acute epidemics and pandemics have been approached, in order to inform strategies to strengthen our collective clinical research preparedness to emerging epidemics [8]. This is, to our knowledge, the first systematic scoping review of solutions to political, economic, administrative, regulatory, logistic, ethical and social (PEARLES) challenges to the design, delivery and implementation of clinical research during emerging epidemics and pandemics.

## Methods

Drawing on PRISMA extension for scoping review guidelines [9], we developed a scoping review protocol in collaboration with researchers with experience in epidemic outbreak research and systematic evidence review methodologies.

### Inclusion criteria

We included published, peer-reviewed quantitative and qualitative studies describing PEARLES challenges and solutions to clinical research responses to epidemics or pandemics identified during previous outbreak responses or through research involving health system stakeholders. We did not exclude reports based on study adesign. We included editorials and other ‘opinion’ articles when these were based on experiences derived from clinical research responses to emerging epidemics or pandemics. Conference abstracts were included as an important source of data not yet published in full [10]. We excluded studies presenting findings only relating to public health responses and not to clinical research. Studies presenting study outcomes without a reflection on challenges and/or solutions were excluded.

### Search and retrieval of studies

The search strategy (Additional file 1) and terms were developed collaboratively with an information specialist who systematically searched six databases (Ovid MEDLINE, Ovid Embase, Global Health, Ovid PsycINFO, Scopus, Epistemonikos) for publications in English from 2008 to July 2018, to include up-to-date information relevant to clinical research responses today. The limits were set to capture recent, relevant clinical research responses to Public Health Emergencies of International Concern (PHEIC), where a clinical research response is vital to forward knowledge into risk factors and optimal clinical care to improve patient outcomes and outbreak control. The search terms were piloted by an information specialist and two reviewers. To ensure the search results were relevant and appropriate, after a review of the pilot search, restrictions were implemented using Boolean operators, before the strategy was finalised [9, 11, 12]. The search strategy was adapted for the Ovid databases to include the relevant thesaurus terms, in addition to searching the title or abstract fields (Table 1). Two reviewers independently screened the title and abstracts of the retrieved articles. If either of the reviewers considered a study potentially eligible, the full-text article was assessed independently for inclusion by two reviewers. Disagreements were resolved by a third reviewer. References were checked for additional potentially eligible studies.

**Table 1:** The search strategy for Scopus and Epistemonikos

(zika* OR zikv* OR ebola* OR “middle east respiratory syndrome*” OR “MERS-CoV” OR h7n9 OR h1n1 OR h5n1 OR nipah OR cholera* OR “yellow fever” OR influenza OR ((outbreak* OR pandemic* OR epidemic*) AND (“infectious disease*” OR “communicable disease*”))) AND (research OR “clinical trial*” OR “vaccin* trial*”) AND (politic* OR economic* OR administrat* OR regulat* OR logistic* OR ethic* OR social* OR cultur* OR behavior* OR behaviour*) AND (barrier* OR bottleneck* OR delay* OR “time delay*” OR expedite* OR solution* OR facilitate*).	

### Data synthesis

One reviewer extracted data from the included studies using standardised forms including information on (1) study characteristics and setting, (2) participants, (3) intervention, (4) type of outcome measures and (5) PEARLES challenges and solutions. A second reviewer checked the extracted data. At the first analysis stage, we coded challenges and solutions according to the PEARLES categories. This showed that although the PEARLES categories were useful for the initial categorisation, there were overlap and interdependencies identified between these categories, especially between political and economic factors and regulatory, logistic and administrative factors and between ethical and social factors. Thus, at the second stage of the analysis, two reviewers identified the sub-themes and actions that emerged under these categories. A risk of bias assessment was not carried out since none of the studies formally evaluated the solutions identified during an epidemic or pandemic. Most of the studies presented challenges encountered while delivering clinical research responses during emerging epidemics and solutions implemented reactively, or solutions identified to address these, without formal evaluation. Lower evidence articles, including opinion pieces, were included to enable capturing the breadth and width of experiences from different settings, to give a voice to research teams delivering clinical research responses in difficult circumstances. Studies covering PEARLES challenges and solutions identified are summarised in the following sections under the interdependent themes that emerged.

## Results

**Fig. 1:**
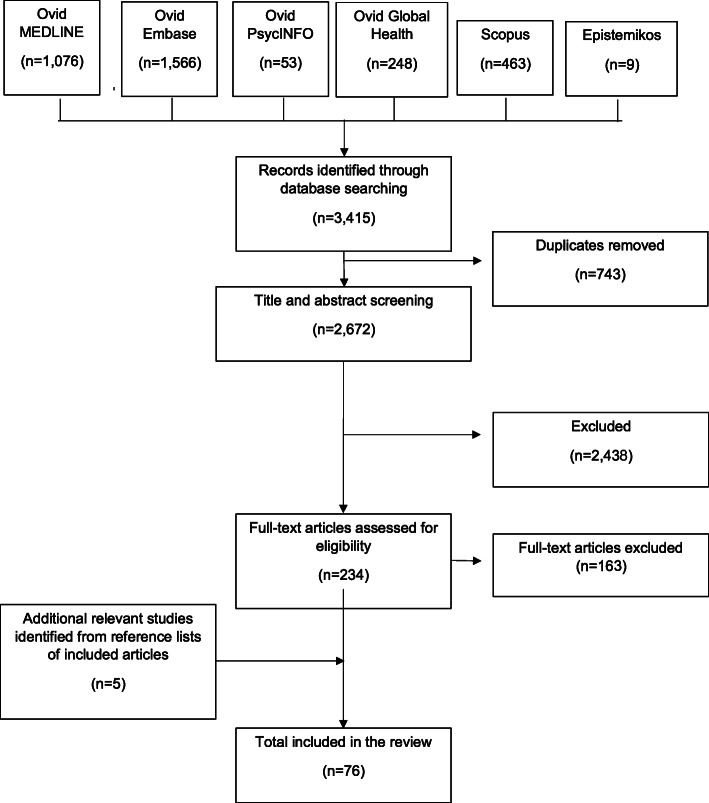
PRISMA diagram

Of the 2673 articles identified through database searching, 234 full-text records were screened for inclusion, 71 of these met the inclusion criteria. Five additional articles were identified from references (Fig. 1).

### Characteristics of included studies

**Fig. 2:**
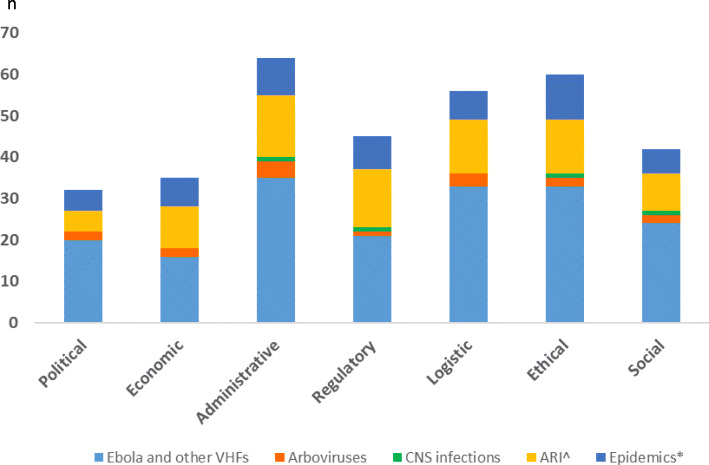
Type of outbreak and PEARLES domains addressed. ^^^Articles focused on influenza, severe acute respiratory infections and pandemics; *Non-specified emergency epidemics. VHF, viral haemorrhagic fevers; Arboviruses, arthropod-borne viruses; CNS, central nervous system; ARI, acute respiratory infections

The study designs of the 76 articles included were systematic review (*n* = 1), narrative reviews (*n* = 19), randomised controlled trials (*n* = 7), other randomised trials (*n* = 8; of which seven were stepped-wedge trials), case-control study (*n* = 1), cohort studies (*n* = 3), cross-sectional study (*n* = 1), time-in-motion study (*n* = 1), qualitative studies (*n* = 15) and editorial, comments or other ‘opinion’ pieces (*n* = 20) (Additional file 2). Most articles presented challenges and solutions identified during the Ebola virus epidemic in 2014 to 2016 and/or during the H1N1 pandemic in 2009. Some articles focused on more than one type of outbreak (Table 2). The studies which used an experimental design (e.g. RCTs) were reporting solutions implemented to deliver the intervention. Most articles related to clinical research responses set in lower-middle-income countries (LMICs) in Africa (*n* = 37), Latin America and the Caribbean (*n* = 3) and Asia (*n* = 1). Thirteen articles related to research responses in higher-income countries (HICs), and 22 articles focused on a global perspective. Most articles addressed more than one PEARLES domains (Fig. 2).

**Table 2:** Study setting and type of outbreaks. Several of the articles focused on a global perspective covered more than one type of outbreak

Outbreak setting	Ebola and other VHFs, *n*	Arboviruses, *n*	CNS infections, *n*	ARI^^^, *n*	Epidemics*, *n*	Total, *n* (%)
Africa	**35**	**–**	**1**	**–**	**1**	**37 (44)**
Asia	**–**	**–**	**–**	**1**	**–**	**1 (1)**
Europe	**1**	**–**	**–**	**7**	**–**	**8 (9)**
Latin America and the Caribbean	**–**	**2**	**–**	**–**	**1**	**3 (4)**
North America	**–**	**–**	**–**	**5**	**–**	**5 (6)**
Global perspective	**13**	**2**	**–**	**6**	**9**	**30 (36)**
Total, *n* (%)	**49 (58)**	**4 (5)**	**1 (1)**	**19 (23)**	**11 (13)**	**84 (100)**

*VHF* viral haemorrhagic fevers, *Arboviruses* arthropod-borne viruses, *CNS* central nervous system, *ARI* acute respiratory infections

*Non-specified emergency epidemics

^^^Includes articles focused on influenza, severe acute respiratory infections and pandemics

### Challenges and solutions

**Fig. 3:**
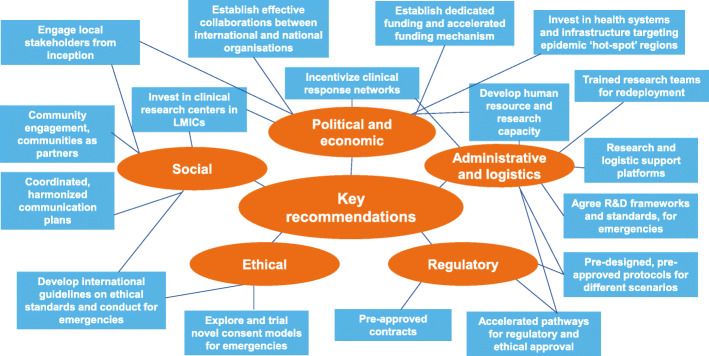
Key cross-cutting actions recommended

There were many solutions identified to address the multiple challenges encountered. These are presented as a narrative summary with illustrative examples from some of the more complex studies. Key actions that emerged are presented in Table 3. Many of these are cross-cutting across domains (Fig. 3).

**Table 3:** Key actions identified

Domain	Key actions identified
Political and economic challenges	Establish effective, coordinated, equitable collaborations between international and national organisations involved in public health emergencies at all levels [1, 3, 7, 13–40]
	Establish dedicated funding and coordinated, accelerated funding mechanisms [1, 13, 15, 19–22, 24, 26, 27, 31–35, 37–39, 41]
	Invest in health systems and infrastructure strengthening, targeting epidemic-prone regions [1–3, 13, 17, 29, 31, 32, 36, 42–44]
	Invest in sustainable clinical research centres and research training [1, 3, 5, 6, 15–17, 20, 22, 29, 31, 33, 38, 44–48]
	Incentivise clinical research response networks [1, 6, 15, 20, 22, 24, 31, 33, 37, 38, 41, 45, 48, 49]
	Engage stakeholders in affected countries from inception [1, 3, 13, 16–19, 22, 26, 29, 31, 32, 36, 38, 40, 44, 50, 51]
Administrative, regulatory and logistic challenges	Develop human resource and research capacity [1, 3–6, 13, 15–17, 20, 22, 29, 31, 33, 36, 38–40, 42–46, 48, 50, 52]
	Train researchers, clinicians and other stakeholders for rapid deployment [1, 36, 38, 41, 53]
	Develop international and national research, administrative and logistics support platforms [1, 3, 4, 18, 20, 29, 32, 42, 43, 45, 47, 50–52, 54–56] with funded coordinating mechanisms [39]
	Agree R&D frameworks and standards for emergencies [2, 5–7, 13, 14, 18, 19, 21, 22, 24, 26, 27, 35, 37, 39–41, 51, 52, 54, 57–60] include national stakeholders [26, 38] and systems for evaluating the impact, regular reviews and updating [19]
	Develop pre-designed and pre-approved study protocols and associated tools for different scenarios [1, 3, 4, 13, 15, 17, 20, 29, 34, 37, 41, 46, 48, 50, 51, 55, 56, 61–63]
	Establish accelerated pathways for regulatory and ethical joint approvals [1, 2, 6, 7, 13, 18, 20, 21, 32, 34, 37, 40, 41, 45, 49, 51, 53, 56, 64–66]
	Set up pre-approved site agreements [4, 15, 38, 45, 46, 48, 61]
	Establish international data and sample sharing agreements and templates [7, 13, 18, 20, 22, 24, 25, 27, 28, 35, 44, 48, 52, 57, 58, 62, 67, 68]
	Establish coordinated, effective internal and external stakeholder communication and communication plans [3, 5, 14, 16, 18, 20, 23, 25, 29, 32, 34, 42, 46, 58, 65, 69–72]
	Ethical emergency publication agreements with focus on timely, open data sharing [2, 22, 38, 52, 68, 73]
Ethical and social challenges	Explore and trial less complex consent models during emergencies [2, 6, 15, 25, 32, 37, 44, 46, 48, 51, 69, 70, 74]
	Develop frameworks for ethical and scientifically robust study designs for various epidemic and pandemic scenarios [6, 13, 14, 17–19, 22, 27, 34, 36, 40, 42, 48, 51, 60, 61, 66, 75–77]
	Develop international guidelines on ethical standards and conduct for emergencies, including inclusion of vulnerable groups [5, 18, 21, 22, 27, 40, 44, 50, 51, 59, 60, 62, 69, 77, 78], and equitable access to care and compensation [44, 59, 77, 79]
	Engage and empower communities and stakeholder from the outset [1, 3, 5, 14, 16, 17, 19, 20, 23, 26, 29, 32, 36, 39, 40, 42, 44, 50, 51, 54, 69, 71, 72, 79, 80]

### Political and economic solutions identified (Table 4)

Political impediments to global collaborative networking and a lack of global coordination of funding and efforts were the key challenges encountered [1, 15, 20, 21, 27, 33, 42]. Delays in mobilising funding [15, 27, 36, 37], with approval sometimes taking longer than the outbreak duration, was a challenge during the H1N1 pandemic in HICs [15, 37] and again during the Ebola outbreak in LMICs [36].

**Table 4:** Solutions to PEARLES challenges encountered

**Political and economic solutions identified**	**Challenges encountered**
**Strengthen global collaborations and coalitions** -Ensure global political awareness of infectious disease threats. -Strengthen collaborations between international organisations, national leaders, public, private and local stakeholders. -WHO to set out overarching research governance framework for research in outbreaks. -Integrate research in international outbreak response. -Ensure interventions are supported by all stakeholders, including national and local stakeholders. -Close collaboration between local and international researchers from research inception, tied to capacity building and be genuinely collaborative. -Research led by national teams. -Invest in national public health research institutes globally, targeting epidemic-prone regions.	**Geopolitical** -Ineffective global coordination and collaboration. -Research not integrated into national outbreak response. -Establishing outbreak as international concern may depend on the ability of LMICs to raise international interest. -Lack of compliance with WHO core capacities to detect, assess, report and respond. -Need for political approval. -Political unrest. -Research priorities dictated by funding bodies. -Lack of communication and engagement between stakeholders.
**Funding** -Establish dedicated funding sources in inter-epidemic times. -Establish international agreements on financial mechanisms for rapid release of funding and for addressing clinical trial liability coverage. -National governments to strengthen investments in preparedness and response. -Coordinate funding to ensure it is rapid and sufficient by using international coalitions and economies of scale. -Ensure sufficient, specific and flexible funding for research staff to avoid healthcare opportunity costs. -Explore industry funding to complement public funding. -Provide appropriate compensation for participation in research.	**Funding** -Insufficient funding resources. -Delays in identifying funding. -Weak funding mechanism and implementation for research in emergencies and neglected and tropical disease. -Funding not mobilisable at sufficient pace. -Limited national health budgets dedicated to research response efforts. -Opportunity costs and competing interests. -Over-reliance on unpaid staff doing research in addition to normal duties, with risk to care, staff and research.
**Health systems and infrastructure** -Strengthen health systems and research capacity. -Strengthen supporting infrastructure, targeting regions vulnerable to epidemics. -Expand critical care resources. -Develop clinical research facilities in predicted ‘hot spot’ regions.	**Health systems and infrastructure** -Limited healthcare systems. -Limited supporting infrastructure, electricity and water supply and technical resources. -Lack of national health research institutes. -Competing interests of resources.
**Administrative, regulatory and logistic solutions identified**	**Challenges encountered**
**Human resources and research capacity** -Ensure capacity to respond to outbreaks across departments, particularly in predictable epidemic-prone regions. -Ensure sufficient support for ethics review boards. -Identify and utilise existing skills and talents, re-deploy existing research staff. -Fund dedicated study teams to avoid additional burden on other staff. -Ensure adequate, sustained research training for staff, particularly during stable periods. -Establish clinical research networks that are incentivised and prepared to respond to outbreaks and politically supported. -Form research response teams, with dedicated research coordinators. -Recruit additional staff from outside of the epidemic area to reduce strain. -Set up mobile research teams to reach large areas. -Improve staff perception of research as a core role of healthcare professionals.	**Human resources and research capacity** -Limited number of staff. -Risk of already scarce staff becoming overwhelmed and additional burden of research activities across services. -Training of staff in research not seen as a priority. -Lack of research coordinators. -Increased workload from study protocol risk negatively affecting patient care. -Difficulties in deploying staff internationally sufficiently rapidly in the context of an outbreak.
**Communication** -Establish direct stakeholder communication channels. -Develop harmonised, coordinated communication activities with shared oversight structures and joint management. -Establish detailed communication and dissemination plans and templates. -Provide continuous updates on research activity to stakeholders as appropriate through a variety of channels. -Set up ‘pandemic champions’ to establish links with sites, to facilitate coordination and to raise awareness.	**Communication** -Data stored in countries other than that affected, disempowers the national team. -LMICs unable to access trial results after the study is completed. -Challenges for LMICs to gain international interest from study results. -Challenging to control the interpretation of research output by the media and political leaders.
**Frameworks** -Establish a normative framework for research and development. -Create frameworks and governance charts for clear decision-making procedures, standard operational procedures and administrative infrastructures. -Prioritise research questions, pre-design study protocols and training materials in advance, ready to be deployed.	**Frameworks** -Lengthy process of planning, formalising and gaining approval of study protocol. -Lack of integrated standards for data collection and infrastructure for data sharing. -Delays in obtaining inter-institutional data sharing agreements. -Time lag for distant reference lab results. -No apparent benefit to those affected to share data. -Delayed data sharing due to academic competition culture.
**Data and sample sharing** -International agencies (e.g. WHO) to establish international data sharing frameworks. -Refinement of international agreements, such as the Declaration of Helsinki to include instructions on how to handle benefit sharing for sponsor and host countries. -Develop templates and platforms for data and sample sharing. -Enhance the value of research output dissemination to all stakeholders through long-term collaboration and health system improvements. -Funding approvals to incorporate agreement on data dissemination. -Establish data sharing ahead of emergencies. -Develop a mechanism to manage intellectual property and data governance. -Establish standard to reduce uncredited secondary analysis to facilitate data sharing. -Ensure global sharing of data with fair distribution. -Enhance the value of the research to each beneficiary through fair dissemination of knowledge, product development, long-term research collaboration, and/or health system improvements.	**Data and sample sharing** -Data collection and sharing on paper records sometimes not possible due to infection control and confidentiality issues. -Loss of control and ownership of data following dissemination. -Data not made available during outbreaks and long delays in publishing data after outbreaks. -Issues around maintaining participant confidentiality when sharing data. -Lack of control of communication. -Confidentiality requirements imposed by commercial entities.
**Publication standards** -Agree open data sharing and publication standards. -A shift in paradigm to a common goal of data sharing rather than publication. -Ensure pre-prints of novel data are available prior to publication. -Develop and use an ‘emergency research pledge’ by journals. -Ensure researchers, including local collaborators, are credited for their work.	**Traditional publication process** -Traditional journal review processes are too slow to inform emergency outbreak response strategies. -Publication authorship imbalances.
**Pathways for regulatory and ethics approvals** -Establish accelerated regulatory pathways and expedited ethical review processes for emergencies. -Establish institutional review boards in epidemic ‘hot spot’ regions. -Enable single portals for applications. -Establish joint ethics review committees. -Enable parallel submission of ethical, financial and scientific approvals. -Develop pre-approved study protocols with agreed acceptable study design modifications. -Develop pre-approved site agreements, between multiple sites and organisations, in geographically strategic regions. -Consideration of the management of bio-samples should be part of the ethical and protocol review.	**Pathways for regulatory and ethics approvals** **-**Delays caused by existing ethical frameworks. -Complex ethic committee forms and inconsistencies between forms. -Variation in REB responses to the same study. -Lack of framework to fast-track vaccine trials or drug testing. -Existing frameworks not fit for emergency research**.** -Lengthy time taken to gain research approvals during epidemics and pandemics. -Requirement of approval from multiple entities (including political) and variations between countries. -Reluctance from national officials to approve trials.
**Drug and vaccine licencing and access** -Form Joint Scientific Advisory and Data Safety Review Committees for all studies linked to a specific intervention or group of interventions. -Global regulatory agencies should collaborate to ensure accelerated licensure strategy. -Contracts with multiple manufacturing and distribution units to improve resilience in the supply of medicines to participating trial site. -Primary role of authorising use of investigational vaccines and drugs should be given to the affected countries.	**Drug and vaccine licencing and access** -Length of time for drugs and vaccines to be approved. -Lack of framework to fast-track vaccine trials and drug testing.
**Research support systems** -Provide sufficient funds for the renovation of study facilities and provide supportive infrastructure. -Strengthen satellite and wireless internet access in epidemic-prone regions. -Use existing infrastructure, e.g. from other disease programmes. -Set up logistical support platforms. -Ensure flexible solutions that can be readily adaptable depending on the context.	**Research support systems** -Rudimentary and overwhelmed healthcare facilities. -Technical resource limitations. -Limited access to freezer storage facilities. -Difficulty reaching remote field sites. -Lack of effective personal protective equipment -Poor safety for staff and participants.
**Ethical and social solutions identified**	**Challenges encountered**
**Standards** -Develop standards for the conduct of research in emergencies, including frameworks for the inclusion of vulnerable groups and appropriate study designs. -Ensure equitable access to best available evidence-based care for all patients, regardless of consent to participate. -Ensure appropriate compensation for participation in research.	**Standards** -Lack of uniform standards for research ethics committees. -Lack of international consensus about research groups’ obligations to provide trial participants health benefits. -Non-transparent ethical approval processes. -Exclusion of pregnant women and children from trials. -Expedited review might pose a risk to patients. -Research perceived as the only way to access care/treatment. -Lack of agreement on appropriate study designs for emergencies.
**Consent methods** -Evaluate alternative consent methods proposed during emergencies. -Ensure consent methods are culturally appropriate.	**Consent methods** -Obtaining complex informed consent, from severely ill patients and from relatives. -Verbal proxy consent contested at later date.
**Community engagement** -Invest in community engagement from inception. -Explore outbreak and community context, and use the findings to inform the study design, set-up, delivery and dissemination. -Ensure protocol and consent forms are consistent with community values and internationally accepted ethical principles. -Build trust through understanding and respect of different cultures. -Ensure study design meets cultural needs. -Manage expectations of all stakeholders. -Facilitate community empowerment. -Provide outreach community information sessions. -Establish community advisory boards. -Use social messaging and informational materials to improve the knowledge and perception of the study and disease and to address rumours. -Ensure effective consultation and communication with affected or at-risk communities, for example, through community liaison teams. -Involve social scientists and medical anthropologists to help understand the concerns and needs of the community. -Translate social science research into practice. -Facilitate relationships between groups with cultural differences.	**Community engagement** -Mistrust, suspicion and rumours around clinical trials, national and international response, sometimes due to local media and other sources. -Poor understanding of how to address rumours. -Fear of the disease. -Cultural perceptions of tissue and blood sampling. -Poor understanding of how to respect different cultures. -Lack of community engagement and poor perception of power dynamics. -Perception of research being unfair.

#### Strengthen collaboration and coordination

A cross-cutting theme identified was the need to strengthen collaboration and coordination between organisations involved in outbreak response at all levels [3, 7, 13–37, 81]. Effective partnerships between countries and international organisations, such as public health, clinical research organisations, non-governmental organisations (NGOs), pharmaceutical companies and regulatory agencies, were described as instrumental for success [3, 14, 16, 19]. International research collaborations should be tied to capacity building and be genuinely collaborative [22], with local stakeholders engaged from inception [1, 3, 13, 16–19, 22, 26, 29, 31, 32, 36, 38, 40, 44, 50, 51]. Support from national governments and local communities was a key enabler [16, 17, 23, 32, 36]. Continuous dialogue, led by governments of affected nations [16, 17], was identified as a facilitator for ensuring politically acceptable prioritisation and resource allocation [36]. This was illustrated by Doe-Anderson et al., where the implementation of a vaccine RCT during an Ebola outbreak was attributed to decisive action by the national government and an effective partnership between the USA and Liberia, with strong leadership from both nations [16]. By employing and training local doctors and scientists and renovation of existing sites for use in the trial, the study also strengthened the research capacity for future trials [16].

#### Establish dedicated funding sources and accelerated funding systems

There were many calls for dedicated funding for emergency research [1, 15, 19, 26, 27, 32, 35, 37], with financial mechanisms for rapid release of funds [1, 15, 20, 21, 26, 27, 33, 42]. Maintaining political awareness of the threat of infectious diseases to global health security (GHS) [35, 63] and an integrated approach to research was recommended to help marshal resources [1, 38]. A coalition of international stakeholders that would provide a global financing facility was suggested, to bring together funds to accelerate and prioritise research and development (R&D) [26, 27] and support R&D for communicable diseases neglected by the commercial market [13]. An example from the UK showed that through an emergency policy activation that allowed expedited funding, approvals and the redeployment of research staff, it was possible to launch a national, multi-site clinical trial within 12 weeks during the 2009 H1N1 pandemic [41, 53].

#### Invest in health systems and infrastructure in epidemic-prone regions

Limited healthcare systems [3, 29, 32, 39] and supporting infrastructure [1–3, 17, 29, 32] and overwhelmed healthcare facilities [31] brought about specific hurdles for delivery of research in LMICs. Investments to strengthen health systems and supporting infrastructure, targeting regions vulnerable to epidemics and pandemics, would facilitate effective responses [1, 2, 13, 29, 36]. Researchers delivering an Ebola vaccine RCT in Sierra Leone illustrated reactive solutions implemented to overcome logistic challenges. To enable recruitment of 8000 healthcare workers, they had to first renovate enrolment sites, laboratories and cold chain facilities and build study facilities and laboratories. Moreover, import freezer equipment and instal satellite-routed internet [29, 42, 43].

### Administrative, regulatory and logistic solutions identified (Table 4)

Administrative and regulatory procedures and limited access to staff with research training were persistent challenges in LMICs and HICs [2, 3, 22, 29, 37, 43, 49, 50, 52]. Medical evacuation insurance requirements [42] and delays in recruiting international staff [38] posed additional challenges in LMICs. This can pose a risk of over-reliance on unpaid staff doing research [22, 29] on top of normal duties, with potential risk to routine patient care [72]. Multiple ethics committees, bureaucratic processes and inconsistency between required documentation were additional hurdles in LMICs and HICs [6, 15, 18, 40, 45, 46, 49, 50, 78], together with staff [42] and trial insurance [69] cover. The longest delays were often experienced in gaining site [48] and/or data sharing agreements, as documented in a time-in-motion study by Rishu et al. (Table 5) [15]. The infrastructure, staff time and an agreed standard required for dissemination of data were also often absent during times of crisis [2, 24, 35, 52] and were further compounded by long delays in institutions establishing data sharing agreements [15]. Some attributed a competitive research culture and a fear of losing power [22, 34] to a reluctance to share data [22, 34, 52, 73].

**Table 5:** Time to initiate an observational study into severe acute respiratory infections in Canada [15]

Set-up time frames	Outbreak	Study setting	Time frame, days, median (IQR)
Overall start-up procedures	H1N1	Canada	335 (128–335)
Site receipt of the protocol to REB submission			73 (30–126)
REB submission to REB approval			43 (13–85)
Protocol receipt to signed data sharing agreement			276 (186–312)

*REB* research ethics board

#### Develop research capacity

The data shows a need to strengthen research capacity [1–3, 13, 29, 36] and invest in training for staff across the board [1, 15, 40, 42, 45, 48] particularly in high-risk regions. Primed clinical research networks globally [6, 24, 31, 37, 48] and a pool of researchers and experts that can be redeployed were recommended [1, 6, 41, 53]. The Platform for European Preparedness Against (Re-) emerging Epidemics was cited as an example of a clinical research network set-up to respond at the outset of an epidemic [20].

#### Frameworks and standards

Internationally agreed frameworks for emergency research to facilitate coordination, focus investments, and to guide implementation of responses are needed. These should identify emerging threats and develop roadmaps to focus R&D investments [19], as well as ethical, regulatory and operational standards for conducting emergency research [1, 7, 13, 19, 27, 35]. The World Health Organization’s (WHO) R&D Blueprint was cited as an example that aims to guide research efforts and set standards for high-priority pathogens [82].

#### Pre-approval and expedited, emergency protocols and frameworks

Pre-approved, pre-positioned study protocols was a key solution recommended to reduce set-up delays [1, 4, 13, 15, 20, 34, 37, 41, 46, 48, 50, 51, 56, 61–63]. These can be strategically developed for a range of syndromes and settings. An international agreement on a financial mechanism to manage clinical trial liability [32] and coverage provided by affected countries [32] was suggested to address delays in gaining insurance cover for all at-risk populations. An article by Lim et al. presented a trial in ‘hibernation’, with full regulatory approvals in place set up in the UK ready to be activated during a future pandemic [46]. Since not all eventualities can be predicted, there is also a need for expedited approval processes for emergencies [1, 2, 6, 7, 13, 18, 32, 34, 41, 45, 53, 54, 56, 64]. There were a couple of examples of expedited approvals (Table 6). A study by Annane et al. concluded that parallel rather than sequential scientific, financial, regulatory and ethics approval, and preparation of study drugs by local pharmacists would have enabled a multi-centre RCT of corticosteroids in ICU patients with H1N1 influenza pneumonia in France to start 1 month earlier, before the peak ‘flu’ wave [6]. Pollard et al. noted that despite expedited processes, the bureaucratic burden was undiminished [53]. Expedited reviews need to be balanced against risk for patients [56]. One article found that double ethical review improved the quality of an Ebola protocol and led to better protection of patients due to the complementarity of the reviews [67]. Ethics committee staff with experience from epidemic research and joint research ethics committees (RECs) with representatives from all affected countries was recommended to facilitate approvals [1, 2, 18, 45] and to protect patient safety [2]. RECs should ensure that protocols are consistent with community values [50], are collaborative and include capacity building [20, 22].

**Table 6:** Expedited ethical review time frames

Expedited ethical review time frames	Outbreak	Study setting	Time frame
Through an accelerated WHO ethical review process, a sub-committee established specifically for review of Ebola studies reviewed protocols in 6 days (max. 15 days) on average during the 2014–2016 outbreak. Barriers causing delays were mainly protocol related [18].	Ebola 2014–2016	West Africa	6 days on average
An H1N1 pandemic vaccine cohort study in the UK in 2009 was through expedited review processes approved by the Oxfordshire Research Ethics Committee within 18 days. Subsequent substantial amendments were approved within 48 h [53].	H1N1 2009	UK	18 days

#### Dissemination

Keeping stakeholders informed of the study progress was cited as a facilitator for engagement and delivery and to prevent misinformation [5, 21, 29, 33]. However, interim data sharing needs to be managed carefully to reduce the risk of misinterpretations [69]. International agencies, such as WHO, are advised to provide a platform for harmonised data sharing [2, 13, 24, 27, 52] and support capacity for data recording in LMICs. In order to encourage data sharing, study approval and funding may be made on the premise of data sharing, and international agreements include guidance on data sharing between sponsors and host countries [52]. To control data sharing, mechanisms need to be in place to ensure that intellectual property, clinical governance and participant confidentiality are maintained [13, 69]. To overcome issues around traditional publication processes, scientific journals should review policies to improve essential data sharing during emergencies [27, 52, 68]. Journals were advised to pledge that data sharing during an emergency would not prejudice later publication [73]. A shift in perspective to a common goal, rather than publications, was called for [2, 52, 73]. There were no data on the implementation of emergency data sharing initiatives identified.

A publication paradigm must change when lives are at risk. Shaw et al [73]

### Ethical and social solutions identified (Table 4)

The temporal and spatial variation in the risk of infection during outbreaks presents not only statistical hurdles but also an ethical challenge for study designs [75]. Previous research responses have highlighted the many challenges in agreeing on simultaneously ethical, scientifically valid and acceptable study designs [18, 36, 42, 51, 59, 64, 66], addressing socially valuable questions [14, 18, 64, 66]. The exclusion of children and pregnant women was an ethical concern during the Ebola outbreak in LMICs [18, 69], compounded by challenges of obtaining complex consent [2, 4, 5, 15, 18, 21, 25, 32, 34, 44–46, 50, 69, 70]. Ethical issues also arose around equity in access to health care for participants and non-participants [44, 50, 59].

There were calls for international standards for the conduct of emergency research [21, 51, 60, 62], including refined definitions of what constitutes research under these circumstances [51], acceptable study designs [14] and simplified consent methods [2, 3, 6, 25, 37, 46, 51, 70, 74]. Some studies explored waivered consent [2, 25, 69, 70, 74] and/or proxy consent [69]. However, gaining proxy consent was also found to be challenging during emergencies [15]. A qualitative study set in Europe found public support for consent waiver for publicly funded, low-risk studies and routinely collected anonymised biological samples for research, and for advanced or verbal consent models for pandemics [70, 74]. An interventional Ebola study set in Guinea reported patient preference for verbal consent [69]. One article highlighted the risks of using non-documented verbal consent [5]. Some, but not all countries, allow research without consent under emergency conditions [2]. Cook et al. argued that a priori and surrogate consent models may be contrary to the public good for research involving critically ill patients during an emerging pandemic and presented a contextually dependent consent model [2]. There was a consensus on recommendations for equitable access to the best available standard of care regardless of consent to participate [29, 32, 50, 59].

#### Community engagement

The need to engage communities is recognised as essential for an effective response. Several articles reported fear and mistrust of international responses as a challenge for research delivery [18, 51, 69, 71, 79]. Integrating all stakeholders into each step of the developing research programme [3, 48, 69], engaging communities as partners [1, 3] and gaining an understanding of power dynamics [34] were facilitators cited to address this. It was also emphasised that interventions need to be culturally sensitive [27, 29, 44, 50, 51, 58] and respond to national as well as global need [36]. Examples of community engagement included regular information sessions [3, 5, 16, 29, 34, 42, 69–71], joint communication plans [20, 29], outreach health promotion teams [69] and formation of community advisory boards [69].

## Discussion

Our findings highlight the many challenges experienced in the planning, conduct and dissemination of clinical research responses during epidemic and pandemic emergencies and the gap in data on our collective capability to respond globally. Although a range of solutions to address these challenges were identified, we did not identify any studies that formally evaluated these during emergencies. We note the consistency with which key recommendations for change have been made across different outbreak experiences. Recommendations made to mitigate challenges experienced during the response to the H1N1 pandemic in 2009 were not globally implemented, and the same issues were documented again during the Ebola response in 2014 to 2016. Most challenges experienced were similar across outbreaks and settings, with additional challenges due to limited healthcare systems and infrastructure encountered in LMICs, and when initiating multi-site responses. The limited data on our capability to respond to a pandemic in LMICs is another area of concern. These hard-learned lessons need firm commitment and action to build clinical research preparedness for future scenarios [83]. Challenges to making progress are varied but include funding shortfalls in the wake of an epidemic as interest wanes [84], structural problems affecting epidemic responses more generally [85] and the absence of clear accountability for action in a policy area that depends on networked responses from many stakeholders across disciplinary and institutional boundaries [83].

This review shows a need to strengthen global collaborations and investments to strengthen research capacity and capability, targeting regions prone to and vulnerable to epidemics in inter-epidemic times. Progress has been made in some areas. The UK NIHR portfolio of ‘hibernating’ pre-approved protocols [86], the global research collaboration for infectious disease preparedness [8] and the WHO blueprint for R&D into high-threat pathogens [87] are steps in the right direction. However, the latter does not include pandemic influenza, and the WHO public health research agenda for influenza does not constitute a strategic plan of action [88].

The Platform for European Preparedness Against (Re-) emerging Epidemics (PREPARE) is an example of a clinical network primed to respond through a suite of active, syndromic studies across Europe [89]. ALERRT and Pandora-ID-Net are similar networks strengthening research capacity across Africa [90, 91]. These could serve as models for similar networks globally. Importantly, these and similar initiatives need to be evaluated and sustained to capitalise on existing investments. Strategic strengthening of local research capability globally will reduce reliance on external agencies to deliver research responses, while ensuring research responses are locally acceptable, ethical in the context and address local needs and interest. Local and regional empowerment may also reduce the risk of unethical studies on vulnerable populations [5]. Work is also needed to review synergies between different initiatives and disease programmes to optimise effectiveness, sustainability and coordination. Integration of research into outbreak response facilitated the further assessment of a candidate Ebola vaccine during the current outbreak in the Democratic Republic of the Congo (DRC), aiding the containment of the outbreak, illustrating the importance of clinical research for outbreak control [92]. A recent RCT of four Ebola treatments in DRC shows that well-planned scientifically and ethically sound clinical research can be delivered during prolonged outbreaks, through coordinated collaborative efforts between a wide range of stakeholders, including frontline staff and patients [93].

A key strength of this study is that it is the first to consider the wide range of challenges to the design and delivery of clinical research during emergency epidemics from a global perspective. The search strategy was comprehensive, spanning multiple databases and incorporating a range of peer-reviewed literature types. Nevertheless, due to the paucity of empirical evidence in this area, we did not carry out a formal quality assessment of included studies. Although findings are limited by restriction to English language publications, most articles addressed challenges to research responses in LMICs. Most studies were set in West Africa during the Ebola outbreak or in Europe and Northern America during the H1N1 pandemic, highlighting the limited information on research preparedness in LMICs and collective preparedness to deliver timely, coordinated research responses to emergency epidemics globally.

## Conclusion

This systematic scoping review shows that potentially effective measures are not being universally implemented despite a good degree of expert consensus on their likely utility. Clinical research communities globally need to evaluate activities, implement solutions identified during previous emergency responses and rehearse and refine outbreak response plans in inter-epidemic times, in collaboration with other organisations involved in outbreak response. Although there may be examples of additional solutions identified in other regions of the world that were not included in this review, we have shown that there is already a substantial body of literature containing valuable experiences and important recommendations. However, without concerted, global action to act and to evaluate those actions in integrated outbreak response plans globally, we may be destined to encounter the same challenges and read about the same suggested solutions in the future. This would mean missed opportunities to forward knowledge into the clinical management of emerging infectious diseases, improve outcomes and strengthen global health security.

## Supplementary information

**Additional file 1.** The search strategy.

**Additional file 2.** An overview of the articles included in the review.

## Data Availability

The full dataset generated and analysed during the current study is available from the corresponding author on reasonable request.

## Abbreviations

ALERRT: African Coalition for Epidemic Research, Response and Training
Arboviruses: Arthropod-borne viruses
ARI: Acute respiratory infections
CNS: Central nervous system
DRC: Democratic Republic of the Congo
EVD: Ebola virus disease
GHS: Global health security
HIC: High-income country
HCWs: Healthcare workers
ICU: Intensive care unit
ID: Infectious disease
LMIC: Low-middle-income country
max.: Maximum
NGOs: Non-governmental organisations
NIHR: National Institute for Health Research
ns: Not specified
PANDORA-ID-NET: Pan-African Network for Rapid Research, Response, Relief and Preparedness for Infectious Disease Epidemics
PEARLES: Political, economic, administrative, regulatory, logistical, ethical and social
PREPARE: Platform for European Preparedness Against (Re-) emerging Epidemics
RCT: Randomised controlled trial
REB: Research ethics board
REC: Research ethics committee
R&D: Research and development
SARS: Severe acute respiratory syndrome
S.E: Southeast
UK: United Kingdom
VHF: Viral haemorrhagic fever
W: West
WHO: World Health Organization
