# Supplementary material for: Addressing challenges for clinical research responses to emerging epidemics and pandemics: a scoping review
Source: BMC Med. 2020 Jun 25;18:190. doi: 10.1186/s12916-020-01624-8 (PMC7315698; doi:10.1186/s12916-020-01624-8)
Supplement: Supplementary file 1 — Additional file 1. The search strategy. [file 12916_2020_1624_MOESM1_ESM.pdf]

Additional file 1. The search strategy

Database: Medline 1946 to present (SEARCH DATE 10/07/18)
Search Strategy:
----
1 exp Disease Outbreaks/ (85621)
2 exp Communicable Diseases/ (32367)
3 1 and 2 (4034)
4 Influenza, Human/ (44616)
5 (zika* or zikv* or ebola* or "middle east respiratory syndrome*" or "MERS-CoV" or H7N9 or H1N1 or H5N1 or nipah or cholera* or "yellow fever").mp. (78613)
6 ((outbreak* or pandemic* or epidemic*) and ("infectious disease*" or "communicable disease*")).ti,ab. (8212)
7 3 or 4 or 5 or 6 (116711)
8 (HIV* or "violence epidemic*" or "opioid epidemic*" or "non-communicable disease*" or "noncommunicable disease*" or diabet* or "chronic condition*" or "cardiac condition*" or obes*).ti,ab. (1036151)
9 7 not 8 (113113)
10 exp Research/ (561779)
11 Research Support as Topic/ (21912)
12 exp Ethics, Research/ (7315)
13 exp clinical study/ (851002)
14 exp clinical studies as topic/ (317481)
15 research.ti,ab. (1218905)
16 ("clinical trial*" or "vaccin* trial*").ti,ab. (313492)
17 10 or 11 or 12 or 13 or 14 or 15 or 16 (2728054)
18 ("public health research" or "vaccin* research*" or "immuni* research*").ti,ab. (3255)
19 17 not 18 (2724836)
20 exp political systems/ or exp politics/ (58631)
21 exp "Organization and Administration"/ (1290522)
22 exp Ethics/ (138142)
23 ethics.sh. (9628)
24 exp Sociology/ (1247343)
25 ((politic* or economic* or administrat* or regulat* or logistic* or ethic* or social* or cultur* or behavior* or behaviour*) and (barrier* or bottleneck* or delay* or "time delay*" or expedite* or solution* or facilitate*)).ti,ab. (420963)
26 20 or 21 or 22 or 23 or 24 or 25 (2754767)
27 9 and 19 and 26 (1535)
28 27 (1535)
29 limit 27 to (english language and yr="2008 -Current") (1076)

Database: Embase 1974 to present (SEARCH DATE 19/07/18)
Search Strategy:
----
1 epidemic/ (95111)
2 communicable disease/ (22250)
3 1 and 2 (2346)
4 Influenza, Human/ (10838)
5 (zika* or zikv* or ebola* or "middle east respiratory syndrome*" or "MERS-CoV" or H7N9 or H1N1 or H5N1 or nipah or cholera* or "yellow fever").mp. (97464)

6 ((outbreak* or pandemic* or epidemic*) and ("infectious disease*" or "communicable disease*")).ti,ab. (10765)
7 3 or 4 or 5 or 6 (117421)
8 (HIV* or "violence epidemic*" or "opioid epidemic*" or "non-communicable disease*" or "noncommunicable disease*" or diabet* or "chronic condition*" or "cardiac condition*" or obes*).ti,ab. (1457631)
9 7 not 8 (112618)
10 exp research/ (728725)
11 research ethics/ (12261)
12 exp clinical study/ (8662446)
13 research.ti,ab. (1628736)
14 ("clinical trial*" or "vaccin* trial*").ti,ab. (443621)
15 10 or 11 or 12 or 13 or 14 (10392570)
16 ("public health research" or "vaccin* research*" or "immuni* research*").ti,ab. (3480)
17 15 not 16 (10389131)
18 exp politics/ (196959)
19 exp political system/ (11446)
20 exp organization/ (579526)
21 exp ethics/ (269619)
22 ethics.sh. (74205)
23 exp sociology/ (93775)
24 ((politic* or economic* or administrat* or regulat* or logistic* or ethic* or social* or cultur* or behavior* or behaviour*) and (barrier* or bottleneck* or delay* or "time delay*" or expedite* or solution* or facilitate*)).ti,ab. (539212)
25 18 or 19 or 20 or 21 or 22 or 23 or 24 (1538291)
26 9 and 17 and 25 (1972)
27 26 (1972)
28 limit 27 to (english language and yr="2008 -Current") (1566)

Database: PsycINFO <1967 to July Week 3 2018> (SEARCH DATE 19/07/18)
Search Strategy:
----
1 epidemics/ (2692)
2 exp infectious disorders/ (57680)
3 1 and 2 (1283)
4 influenza/ (1207)
5 (zika* or zikv* or ebola* or "middle east respiratory syndrome*" or "MERS-CoV" or H7N9 or H1N1 or H5N1 or nipah or cholera* or "yellow fever").mp. (1534)
6 ((outbreak* or pandemic* or epidemic*) and ("infectious disease*" or "communicable disease*")).ti,ab. (512)
7 3 or 4 or 5 or 6 (3888)
8 (HIV* or "violence epidemic*" or "opioid epidemic*" or "non-communicable disease*" or "noncommunicable disease*" or diabet* or "chronic condition*" or "cardiac condition*" or obes*).ti,ab. (109531)
9 7 not 8 (2756)
10 exp experimentation/ (72665)
11 experimental ethics/ (3905)
12 clinical trials/ (10959)
13 research.ti,ab. (885730)
14 ("clinical trial*" or "vaccin* trial*").ti,ab. (28503)
15 10 or 11 or 12 or 13 or 14 (923959)

16 ("public health research" or "vaccin* research*" or "immuni* research*").ti,ab. (652)
17 15 not 16 (923308)
18 exp politics/ (35923)
19 exp organizations/ (68684)
20 exp ethics/ (40230)
21 ethics.sh. (12595)
22 sociology/ (11078)
23 ((politic* or economic* or administrat* or regulat* or logistic* or ethic* or social* or cultur* or behavior* or behaviour*) and (barrier* or bottleneck* or delay* or "time delay*" or expedite* or solution* or facilitate*)).ti,ab. (132836)
24 18 or 19 or 20 or 21 or 22 or 23 (276352)
25 9 and 17 and 24 (61)
26 25 (61)
27 limit 26 to (english language and yr="2008 -Current") (53)

Database: Global Health <1973 to 2018 Week 27> (SEARCH DATE 19/07/18)
Search Strategy:
----
1 outbreaks/ (30757)
2 exp infectious diseases/ (43865)
3 1 and 2 (3331)
4 influenza viruses.sh. (16375)
5 (zika* or zikv* or ebola* or "middle east respiratory syndrome*" or "MERS-CoV" or H7N9 or H1N1 or H5N1 or nipah or cholera* or "yellow fever").mp. (34780)
6 ((outbreak* or pandemic* or epidemic*) and ("infectious disease*" or "communicable disease*")).ti,ab. (5838)
7 3 or 4 or 5 or 6 (52127)
8 (HIV* or "violence epidemic*" or "opioid epidemic*" or "non-communicable disease*" or "noncommunicable disease*" or diabet* or "chronic condition*" or "cardiac condition*" or obes*).ti,ab. (337951)
9 7 not 8 (49562)
10 exp research/ (42616)
11 exp clinical trials/ (46045)
12 research.ti,ab. (221414)
13 ("clinical trial*" or "vaccin* trial*").ti,ab. (35395)
14 10 or 11 or 12 or 13 (303364)
15 ("public health research" or "vaccin* research*" or "immuni* research*").ti,ab. (1632)
16 14 not 15 (301747)
17 politics/ or political systems/ (2282)
18 exp organizations/ (26010)
19 exp ethics/ (5731)
20 ethics.sh. (5212)
21 exp sociology/ (17622)
22 ((politic* or economic* or administrat* or regulat* or logistic* or ethic* or social* or cultur* or behavior* or behaviour*) and (barrier* or bottleneck* or delay* or "time delay*" or expedite* or solution* or facilitate*)).ti,ab. (56772)
23 17 or 18 or 19 or 20 or 21 or 22 (104001)
24 9 and 16 and 23 (324)
25 24 (324)
26 limit 25 to (english language and yr="2008 -Current") (248)

Scopus (SEARCH DATE 19/07/18)

1. TITLE-ABS-KEY (zika* OR zikv* OR ebola* OR "middle east respiratory syndrome*" OR "MERS-CoV" OR h7n9 OR h1n1 OR h5n1 OR nipah OR cholera* OR "yellow fever" OR influenza)
2. TITLE-ABS-KEY (((outbreak* OR pandemic* OR epidemic*) AND ("infectious disease*" OR "communicable disease*")))
3. #1 AND #2
4. (TITLE (hiv* OR "violence epidemic*" OR "opioid epidemic*" OR "non-communicable disease*" OR "noncommunicable disease*" OR diabet* OR "chronic condition*" OR "cardiac condition*" OR obes*) OR ABS (hiv* OR "violence epidemic*" OR "opioid epidemic*" OR "non-communicable disease*" OR "noncommunicable disease*" OR diabet* OR "chronic condition*" OR "cardiac condition*" OR obes*))
5. #3 AND NOT #4
6. (TITLE (research OR "clinical trial*" OR "vaccin* trial*") OR ABS (research OR "clinical trial*" OR "vaccin* trial*"))
7. (TITLE ("public health research" OR "vaccin* research*" OR "immuni* research*") OR ABS ("public health research" OR "vaccin* research*" OR "immuni* research*"))
8. #6 AND NOT #7
9. TITLE-ABS-KEY (((politic* OR economic* OR administrat* OR regulat* OR logistic* OR ethic* OR social* OR cultur* OR behavior* OR behaviour*) AND (barrier* OR bottleneck* OR delay* OR "time delay*" OR expedite* OR solution* OR facilitate*)))
10. #5 AND #8 AND #9
11. 10 AND (LIMIT-TO (PUBYEAR , 2018) OR LIMIT-TO (PUBYEAR , 2017) OR LIMIT-TO (PUBYEAR , 2016) OR LIMIT-TO (PUBYEAR , 2015) OR LIMIT-TO (PUBYEAR , 2014) OR LIMIT-TO (PUBYEAR , 2013) OR LIMIT-TO (PUBYEAR , 2012) OR LIMIT-TO (PUBYEAR , 2011) OR LIMIT-TO (PUBYEAR , 2010) OR LIMIT-TO (PUBYEAR , 2009) OR LIMIT-TO (PUBYEAR , 2008)) AND (LIMIT-TO (LANGUAGE , "English"))

Epistemikos [Available at: https://www.epistemonikos.org] (SEARCH DATE 19/07/18)
TITLE/ABSTRACT: (zika* OR zikv* OR ebola* OR "middle east respiratory syndrome*" OR "MERS-CoV" OR h7n9 OR h1n1 OR h5n1 OR nipah OR cholera* OR "yellow fever" OR influenza OR ((outbreak* OR pandemic* OR epidemic*) AND ("infectious disease*" OR "communicable disease*"))) AND (research OR "clinical trial*" OR "vaccin* trial*") AND ((politic* OR economic* OR administrat* OR regulat* OR logistic* OR ethic* OR social* OR cultur* OR behavior* OR behaviour*) AND (barrier* OR bottleneck* OR delay* OR "time delay*" OR expedite* OR solution* OR facilitate*)). Limited to publications from 2008-current date

Search Results

Ovid Medline	1076
Ovid Embase	1566
Ovid PsycINFO	53
Ovid Global Health	248
Scopus	463
Epistemikos	9
Total	3415
Total after deduplication	2673
