# Supplementary material for: Addressing challenges for clinical research responses to emerging epidemics and pandemics: a scoping review
Source: BMC Med. 2020 Jun 25;18:190. doi: 10.1186/s12916-020-01624-8 (PMC7315698; doi:10.1186/s12916-020-01624-8)
Supplement: Supplementary file 2 — Additional file 2. An overview of the articles included in the review. [file 12916_2020_1624_MOESM2_ESM.pdf]

Additional file 2. An overview of the articles included in the review

Author/s	Title	Source of information	Population	Study setting	Outbreak	Solutions identified or recommended by PEARL(E)S domain
Alirol E et al. 2017	Ethics review of studies during public health emergencies - the experience of the WHO ethics review committee during the Ebola virus disease epidemic (17)	Qualitative study	Paediatric and adults (incl. pregnant women)	W. Africa - Guinea, Liberia, Sierra Leone	Ebola	P, A, R, L, (E), S
Annane D et al. 2012	Designing and conducting a randomized trial for pandemic critical illness: the 2009 H1N1 influenza pandemic. (6)	RCT	>15 years (pregnant women excluded)	Europe - France	H1N1	A, R, L, (E)
Bausch DG et al. 2008	Treatment of Marburg and Ebola haemorrhagic fevers: a strategy for testing new drugs and vaccines under outbreak conditions. (3)	Narrative review	ns	W. Africa	Lassa fever and other filoviruses	P, E, A, R, L, (E), S
Bellan SE et al. 2015	Statistical power and validity of Ebola vaccine trials in Sierra Leone: a simulation study of trial design and analysis. (74)	RCT and Stepped-wedge cluster trial modelling	Populations at risk of EVD	W. Africa - Sierra Leone	Ebola	L, (E)
Berry SM et al. 2016	A response adaptive randomisation platform trial for efficient evaluation of Ebola virus treatments: a model for pandemic response. (49)	Adaptive RCT	Paediatrics and adults (incl. pregnant women)	W. Africa - Sierra Leone	Ebola	A, R, L, (E), S
Burns KE et al. 2013	Participation of ICUs in critical care pandemic research: a province wide, cross-sectional survey. (36)	Cross-sectional study	Research coordinators and ICU administrators	Canada	H1N1	E, A, R, L, (E)
Calain P et al. 2009	Research ethics and international epidemic response: The case of Ebola and Marburg haemorrhagic fevers. (50)	Narrative review	ns	Global	Ebola, Marburg, SARS and H5N1	A, R,(E), S
Canario Guzman JA et al. 2017	Ethical challenges for international collaborative research partnerships in the context of the Zika outbreak in the Dominican Republic: a qualitative case study. (21)	Qualitative case study	Public sector, NGOs, professionals and community stakeholders	Caribbean - Dominican Republic	Zika	A, L, (E) S
Carazo Perez S et al. 2017	Challenges in preparing and implementing a clinical trial at field level in an Ebola emergency: A case study in Guinea, West Africa. (68)	Qualitative case study	> 1-year olds	W. Africa - Guinea	Ebola	A, R, L, (E), S
Carter R et al. 2018	Implementing a Multisite Clinical Trial in the Midst of an Ebola Outbreak: Lessons Learned from the Sierra Leone Trial to Introduce a Vaccine Against Ebola. (28)	Stepped-wedge, randomized trial (linked with Widdicombe et al. 2016, Idriss et al. 2017)	Healthcare workers	W. Africa - Sierra Leone	Ebola	P, E, A, L, (E), S
Cavaleri M et al. 2016	A viewpoint on European Medicines Agency experience with investigational medicinal products for Ebola. (63)	Opinion piece	ns	Europe	Ebola	P, A, R, L, (E), S
Chretien JP et al. 2016	Make data sharing routine to prepare for public health emergencies. (51)	Opinion piece	ns	Global	ID Public health emergencies	E, A, R, L, (E) , S
Cook D et al. 2010	Clinical research ethics for critically ill patients: a pandemic proposal. (2)	Opinion piece	ns	Global	H1N1	A, R, L, (E), S
Coulibaly F et al. 2017	"Conducting clinical trials in crisis settings, 2012 military coup in Mali and the Ebola virus outbreak in 2014 in West Africa. (22)	Qualitative study	ns	W. Africa - Mali	Ebola	P,L, S
Crowcroft NS et al. 2014	The ethics of sharing preliminary research findings during public health emergencies: a case study from the 2009 influenza pandemic. (57)	Qualitative case study	ns	Canada	Influenza	R, S
De Crop M et al. 2016	Multiple ethical review in North–South collaborative research: the experience of the Ebola-Tx trial in Guinea. (64)	Qualitative study	ns	W. Africa - Guinea	Ebola	A, R, L,(E)
Doe-Anderson J et al. 2016	Beating the odds: successful establishment of a phase II/III clinical research trial in resource-poor Liberia during the largest-ever Ebola outbreak. (15)	RCT (linked with Larson et al. 2017)	Healthy adults, HCWs, ambulance staff, surveillance and burial staff.	W. Africa - Liberia	Ebola	P, A, L, S

Additional file 2. An overview of the articles included in the review

			(Excl. pregnant women, lactating mothers, children)			
Dunning J et al. 2016	Experimental Treatment of Ebola Virus Disease with Brincidofovir. (56)	Cohort single-arm trial (linked with Rojek et al. 2018)	Paediatric (> 2 months old) and adults	W. Africa - Liberia	Ebola	L, (E)
Ebola ca Suffit Ring Vaccination Trial Consortium 2015	The ring vaccination trial: a novel cluster randomised controlled trial design to evaluate vaccine efficacy and effectiveness during outbreaks, with special reference to Ebola. (46)	Stepped-wedge cluster randomised trial	People newly diagnosed with EVD and contacts	W. Africa - Guinea	Ebola	A, L, (E)
Ellenberg SS et al. 2018	Rigorous clinical trial design in public health emergencies is essential. (13)	Narrative review	ns	W. Africa	Ebola	P, (E), S
Enria L et al. 2016	Power, fairness and trust: understanding and engaging with vaccine trial participants and communities in the setting up the EBOVAC-Salone vaccine trial in Sierra Leone. (71)	Qualitative study (linked with Smout et al. 2016, Mooney et al. 2018)	Clinical trial participants and other stakeholders	W. Africa - Sierra Leone	Ebola	A, L, (E), S
Ezeome ER et al. 2010	Ethical problems in conducting research in acute epidemics: the Pfizer meningitis study in Nigeria as an illustration. (5)	Narrative review	ns	W. Africa - Nigeria	Meningococcal meningitis	A, R, (E), S
Folayan MO et al. 2015	Ethical considerations in the conduct of research on therapies for the prevention and treatment of Ebola virus disease in developing countries. (16)	Narrative review	ns	W. Africa	Ebola	P, A, L, (E)
Fowler RA et al. 2010	Early observational research and registries during the 2009-2010 influenza a pandemic. (54)	Narrative review	ns	Global	H1N1	E, A, L
Gobat N et al. 2016	Public attitudes towards research participation during an infectious disease pandemic: a qualitative study across four European countries. (69)	Qualitative study (linked with Gobat et al. 2018)	Adults	Europe - Belgium, Poland, Spain, UK	Pandemic	(E), S
Gobat NH et al. 2018	Talking to the people that really matter about their participation in pandemic clinical research: A qualitative study in four European countries. (73)	Qualitative study (linked with Gobat et al. 2016)	Adults	Europe - Belgium, Poland, Spain, UK	Pandemic	A, L, (E)
Gostin LO et al. 2016	Toward a common secure future: four global commissions in the wake of Ebola. (12)	Narrative review	ns	Global	Ebola	P, E, R, L
Henao-Restrepo et al. 2016	On a path to accelerate access to Ebola vaccines: The WHO's research and development efforts during the 2014–2016 Ebola epidemic in West Africa. (18)	Narrative review	ns	W. Africa	Ebola	P, E, A, R, L, (E), S
Heymann DL et al. 2015	Ebola vaccines: keep the clinical trial protocols on the shelf and ready to roll out. (53)	Opinion piece	ns	Global	Ebola	E, R, L, (E), S
Higgs ES et al. 2008	The Southeast Asian Influenza Clinical Research Network: Development and challenges for a new multilateral research endeavour. (29)	Narrative review	ns	S.E. Asia – Thailand and Vietnam	Influenza	P, A, R, (E)
Hurst DJ et al. 2017	Benefit Sharing in a Global Context: Working Towards Solutions for Implementation. (27)	Narrative review	ns	Global	Pandemic	P, E
Idriss A et al. 2017	Sierra Leone trial to introduce a vaccine against Ebola (STRIVE): Implementation challenges, successes and lessons learned. (42)	Stepped-wedge, randomised trial (linked with Widdicombe et al. 2016, Carter et al. 2018)	Healthcare and frontline response workers	W. Africa - Sierra Leone	Ebola	A, L
Johansson MA et al. 2018	Preprints: An underutilized mechanism to accelerate outbreak science. (67)	Opinion piece	ns	Global	Ebola and Zika	A
Keusch GT et al. 2017	Clinical trials during epidemics. (25)	Opinion piece	ns	W. Africa - Guinea, Sierra Leone, Liberia	Ebola	P, E, A, R, (E), S
Kho ME et al. 2011	Costs of clinical research preparation for the H1N1 pandemic in Canada: a single centre, multi-site analysis. (48)	Opinion piece	ns	Canada	H1N1	R

Additional file 2. An overview of the articles included in the review

Kieny MP et al. 2016	Regulatory policy for research and development of vaccines for public health emergencies. (7)	Opinion piece	ns	Global	ID Public health emergencies (incl. Ebola)	P, A, R
Kieny MP et al. 2017	WHO R&D Blueprint: a global coordination mechanism for R&D preparedness. (81)	Opinion piece	ns	Global	Ebola	A, R
Koita OA et al. 2016	Clinical research and the training of host country investigators: essential health priorities for disease-endemic regions. (30)	Opinion piece	ns	W. Africa - Mali	ID Public health epidemics (incl. Ebola)	P, E, A, L, (E)
Kombe et al. 2016	Taking the bull by the horns: Ethical considerations in the design and implementation of an Ebola virus therapy trial. (58)	Narrative review	ns	W. Africa	Ebola	A, L, (E)
Kummervold et al. 2017	Controversial Ebola vaccine trials in Ghana: a thematic analysis of critiques and rebuttals in digital news. (78)	Qualitative study	ns	W. Africa - Ghana	Ebola	E
Kurz X et al. 2017	The ADVANCE Code of Conduct for collaborative vaccine studies. (61)	Qualitative study	ns	Global	ID Public health emergencies (incl. H1N1)	A, R, (E)
Lang T 2015	Embed research in outbreak response. (37)	Opinion piece	ns	W. Africa	Ebola	A, L
Lanini S et al. 2015	Are adaptive randomised trials or non-randomised studies the best way to address the Ebola outbreak in west Africa? (76)	Opinion piece	ns	W. Africa - Guinea, Liberia, Sierra Leone	Ebola	(E)
Larson GS et al. 2017	Conventional Wisdom versus Actual Outcomes: Challenges in the Conduct of an Ebola Vaccine Trial in Liberia during the International Public Health Emergency. (31)	RCT (linked with Doe-Anderson et al. 2016)	Adults >18 years old (excl. pregnant and breastfeeding women)	W. Africa - Liberia	Ebola	P, E, A, R, L, S
Levine AC 2016	Academics are from Mars; humanitarians are from Venus: Finding common ground to improve research during humanitarian emergencies. (38)	Opinion piece	ns	W. Africa - Liberia	Ebola	E, A, L, S
Lie et al. 2017	The Guinea phase III Ebola vaccine trial: lessons for research ethics review in public health emergencies. (55)	Narrative review	ns	Global	Ebola	A, R, L, (E)
Lim WS et al. 2015	Blinded randomised controlled trial of low-dose Adjuvant Steroids in Adults admitted to hospital with Pandemic influenza (ASAP): a trial 'in hibernation', ready for rapid activation. (45)	RCT	Adults ≥ 16-year olds	Europe - UK	Influenza/pandemic	P, A, R, L,(E), S
Lipsitch M et al. 2017	Improving vaccine trials in infectious disease emergencies.(60)	Narrative review	ns	Global	Ebola	A, R, (E)
Lurie et al. 2013	Research as a Part of Public Health Emergency Response. (1)	Opinion piece	ns	Global	ID public health emergency (incl. H1N1 pandemic)	E, A, L, (E), S
Matthiessen et al. 2016	Coordinating funding in public health emergencies. (23)	Opinion piece	ns	Global	Ebola, Zika and yellow fever	P, E, A, L
Molyneux M 2017	New ethical considerations in vaccine trials. (43)	Opinion piece	ns	Global	ID emergencies	A, R, (E), S
Moon S et al. 2015	Will Ebola change the game? Ten essential reforms before the next pandemic. the report of the Harvard-LSHTM Independent Panel on the Global Response to Ebola. (26)	Qualitative study	Adult expert panel	Global	Ebola	P, E, A, R, L, (E)
Mooney et al. 2018	EBOVAC-Salone: Lessons learned from implementing an Ebola vaccine trial in an Ebola-affected country. (39)	Stepped-wedge randomised trial (linked with Enria et al. 2016, Smout et al. 2016)	Adults	W. Africa - Sierra Leone	Ebola	P, A, (E), S
Pollard et al. 2009	Expediting clinical trials in a pandemic. (52)	Cohort trial	6 months to 12-year olds	Europe - UK	H1N1	E, A, R, L, (E), S

Additional file 2. An overview of the articles included in the review

Pollock NR et al. 2017	Evaluating novel diagnostics in an outbreak setting: Lessons learned from Ebola. (20)	Opinion piece	ns	W. Africa - Guinea, Liberia, Sierra Leone	Ebola	P, E, A, (E), S
Ravinetto R et al. 2015	Multiple ethical review in North-South collaborative research in an emergency context: The experience of the Ebola-Tx trial. (66)	Qualitative study	ns	W. Africa - Guinea	Ebola	A, (E)
Richardson T et al. 2017	A systematic review of Ebola treatment trials to assess the extent to which they adhere to ethical guidelines. (59)	Systematic review	ns	W. Africa - Guinea, Liberia, Sierra Leone	Ebola	A, (E)
Rid et al. 2016	Ethical rationale for the Ebola "ring vaccination" trial design. (65)	Narrative review	ns	W. Africa - Guinea	Ebola	L, (E)
Rishu AH et al. 2017	Time required to initiate outbreak and pandemic observational research. (14)	Time-in-motion study	Paediatric and adults in ICUs	Canada	Outbreak/pandemic	P, E, A, R, L, (E)
Rojek AM et al. 2018	Regulatory and operational complexities of conducting a clinical treatment trial during an Ebola virus disease epidemic. (4)	Cohort – single-arm trial (linked with Dunning et al. 2016	Not stated	W. Africa - Sierra Leone	Ebola	A, R, L
Rojek AM et al. 2016	Modernising Epidemic Science: enabling patient-centred research during epidemics. (47)	Narrative review	ns	Global	Epidemics (incl. Ebola)	E, A, L, (E), S
Ruiz-Palacios G et al. 2011	La red: The formation of an emerging infectious diseases clinical research network in Mexico. (32)	Narrative review	ns	Latin America - Mexico	Epidemics	P, E, A, R, L, (E)
Scheifele DW et al. 2011	Strategies for successful rapid trials of influenza vaccine. (44)	Randomised, blinded cross-over trial	Adults	Canada	Influenza	E, A, R, L
Schieffelin J et al. 2016	Clinical Validation Trial of a Diagnostic for Ebola Zaire Antigen Detection: Design Rationale and Challenges to Implementation. (24)	Case-control study.	EVD cases	W. Africa - Sierra Leone	Ebola	P, R, L, (E)
Schopper D et al. 2017	Research ethics governance in times of Ebola. (77)	Opinion piece	ns	W. Africa - Guinea, Libera, Sierra Leone	Ebola	(E)
Sethi N et al. 2016	Conducting research in the context of global health emergencies: identifying key ethical and governance issues. (33)	Narrative review	ns	Global	Public health emergencies (incl. epidemics)	P, E, R, L, (E), S
Shaw D et al. 2017	Publication ethics in public health emergencies. (72)	Opinion piece	ns	Global	Public health emergencies (incl. epidemics)	R, (E), S
Shrivastava SR et al. 2017	Strengthening research and development activities to effectively contain the epidemics of infectious diseases: World health organization. (34)	Opinion piece	ns	Global	Epidemics	P, E, A, R, L, (E)
Smout EM et al. 2016	Implementing a novel community engagement system during a clinical trial of a candidate Ebola vaccine within an outbreak setting. (70)	Qualitative study (linked with Enria 2016, Mooney 2018)	ns	W. Africa - Sierra Leone	Ebola	S
Tengbeh AF et al. 2018	"We are the heroes because we are ready to die for this country": Participants' decision-making and grounded ethics in an Ebola vaccine clinical trial. (79)	Qualitative study	Adults, with a focus on HCWs.	W. Africa - Sierra Leone	Ebola	S
Thielman NM et al. 2016	Ebola clinical trials: Five lessons learned and a way forward. (35)	Opinion piece	ns	W. Africa – Liberia , Sierra Leone	Ebola	P, E, A, L, S
Vandebosch A et al. 2016	Simulation-guided phase 3 trial design to evaluate vaccine effectiveness to prevent Ebola virus disease infection: Statistical considerations, design rationale, and challenges. (75)	Stepped-wedge cluster randomised trial modelling	ns	Global	Ebola	R, L, (E), S
Walley T et al. 2010	Research funding in a pandemic. (40)	Narrative review	ns	Europe - UK	H1N1	E, A, R, L, (E), S
Whitham D et al. 2013	Randomised trials during a public health crisis, such as pandemic flu: A case study for a model of 'off the shelf' ready-to-go trials. (62)	RCT	Adults	Europe - UK	Influenza	P, R

Additional file 2. An overview of the articles included in the review

Widdowson MA et al. 2016	Implementing an Ebola Vaccine Study - Sierra Leone. (41)	Stepped-wedge randomised trial (linked to Idriss 2017, Carter 2018)	HCWs and other Ebola frontline workers	W. Africa - Sierra Leone	Ebola	E, A, R, L, (E), S
Wilder-Smith A et al. 2017	ZikaPLAN: Zika Preparedness Latin American Network. (19)	Narrative review	ns	Latin Americas	Zika	P, E, A, R, L, (E), S

Legend keys: ns: not specified. Abbreviations: EVD: Ebola virus disease, HCWs: healthcare workers, ICU: Intensive care unit, ID: Infectious disease, NGOs: non-governmental organisations, RCT: randomised controlled trial, R&D: research and development, SARS: severe acute respiratory syndrome, UK: United Kingdom, WHO: World Health Organisation
